# Evaluation of Acute and Sub-Acute Toxicity, Oxidative Stress and Molecular Docking of Two Nitrofuranyl Amides as Promising Anti-Tuberculosis Agents

**DOI:** 10.3390/biom13081174

**Published:** 2023-07-28

**Authors:** Simeon Dimitrov, Ivaylo Slavchev, Rumyana Simeonova, Milka Mileva, Tania Pencheva, Stanislav Philipov, Almira Georgieva, Elina Tsvetanova, Yoanna Teneva, Nadezhda Rimpova, Georgi Dobrikov, Violeta Valcheva

**Affiliations:** 1 The Stephan Angeloff Institute of Microbiology, Bulgarian Academy of Sciences, 1113 Sofia, Bulgaria; simo_vets@abv.bg (S.D.); milkamileva@gmail.com (M.M.); almirageorgieva@gmail.com (A.G.); elinaroum@yahoo.com (E.T.); 2 Institute of Organic Chemistry with Centre of Phytochemistry, Bulgarian Academy of Sciences, 1113 Sofia, Bulgaria; ivaylo.slavchev@orgchm.bas.bg (I.S.); georgi.dobrikov@orgchm.bas.bg (G.D.); 3 Department of Pharmacology, Pharmacotherapy, and Toxicology, Faculty of Pharmacy, Medical University of Sofia, 1000 Sofia, Bulgaria; rvitanska@gmail.com (R.S.); yoanna.koedzhikova@gmail.com (Y.T.); 4 Institute of Biophysics and Biomedical Engineering, Bulgarian Academy of Sciences, 1113 Sofia, Bulgaria; tania.pencheva@biomed.bas.bg; 5 Department of Human Anatomy, Histology, General and Clinical Pathology and Forensic Medicine, Faculty of Medicine, Sofia University “St. Kliment Ohridski”, 1407 Sofia, Bulgaria; stanislav_philipov@abv.bg; 6 Institute of Neurobiology, Bulgarian Academy of Sciences, 1113 Sofia, Bulgaria; 7 Department of Paediatrics, University Children’s Hospital, Medical University of Sofia, 1431 Sofia, Bulgaria; nadiarimpova@abv.bg

**Keywords:** nitrofuranyl amides, acute and sub-acute toxicity, markers of oxidative stress, molecular docking, pathomorphological evaluation

## Abstract

Tuberculosis (TB) remains a widespread infectious disease and one of the top 10 causes of death worldwide. Nevertheless, despite significant advances in the development of new drugs against tuberculosis, many therapies and preventive measures do not lead to the expected favorable health results for various reasons. The aim of this study was to evaluate the acute and sub-acute toxicity and oxidative stress of two selected nitrofuranyl amides with high in vitro antimycobacterial activity. In addition, molecular docking studies were performed on both compounds to elucidate the possibilities for further development of new anti-tuberculosis candidates with improved efficacy, selectivity, and pharmacological parameters. Acute toxicity tests showed that no changes were observed in the skin, coat, eyes, mucous membranes, secretions, and vegetative activity in mice. The histological findings include features consistent with normal histological architecture without being associated with concomitant pathological conditions. The observed oxidative stress markers indicated that the studied compounds disturbed the oxidative balance in the mouse liver. Based on the molecular docking, compound **DO-190** showed preferable binding energies compared to **DO-209** in three out of four targets, while both compounds showed promising protein–ligand interactions. Thus, both studied compounds displayed promising activity with low toxicity and can be considered for further evaluation and/or lead optimization.

## 1. Introduction

Tuberculosis (TB) remains a socially significant infectious disease worldwide. Some new anti-tuberculosis drugs have been approved for clinical use recently [1]. However, it seems that they demonstrate many side effects, despite their enhanced efficiency and advantages over classic first-line anti-TB drugs. For example, bedaquiline, which came into clinical practice, demonstrated an adverse effect on heart rhythm [2,3]. This was omitted by the pharmaceutical companies during the extensive clinical trials in earlier years. All this suggests that universal and ideal anti-tuberculosis chemotherapeutics do not exist and the search for new drugs remains relevant. A good approach to finding them faster is to search among classes of compounds that have already shown antibacterial activity.

Nitrofuranes have been known as antibacterials for decades. In recent years a limited number of nitrofuranyl compounds were tested in vitro and in vivo as potential anti-tubercular agents recent [4,5,6,7,8,9,10,11].

However, only a few studies investigated their pharmacology, molecular docking, toxicology, and possible mechanisms of action [7,9,10,12,13]. Hevener et al. also performed quantitative structure-activity relationship (QSAR) analysis of a large number of nitrofuranes and explained why nitrofuranes (and particularly piperazine-containing nitrofuranes) are perspective anti-tubercular agents. [8]. In our recent study, we performed the synthesis of small series of new nitrofuranyl amides [14]. We investigated their anti-TB activity and primary genetic response of mycobacteria (for selected single piperazine-containing amide) through whole-genome sequencing (WGS) of spontaneous resistant mutants.

The effectiveness of TB drugs in the presence of liver damage should take into account the benefit/risk ratio of the TB treatment course. Extrapulmonary organ involvement has been associated with the incidence of anti-TB drug-induced hepatotoxicity. Extrapulmonary lesions in TB patients often include serious lesions in the liver. Hepatotoxicity studies demonstrate the formation and accumulation of reactive metabolites, such as lipid peroxidation products, e.g., malondialdehyde. Such adducts inhibit cellular antioxidant defense mechanisms—enzymatic and non-enzymatic, and as a serious consequence of the development of oxidative stress, they can modulate cell death of hepatocytes [15]. Most first-line anti-TB drugs are lipophilic, and their biotransformation involves their conversion to water-soluble compounds. Cytochrome P450-dependent phase I monooxygenase enzyme systems, involving oxidation, reduction, or hydrolysis processes are responsible for this process. High levels of reactive metabolite formation could be due to high levels or increased activity of these enzymes [16,17].

There is evidence that when isoniazid and rifampicin are metabolized mainly in the liver, a large amount of intracellular glutathione is consumed during the metabolism of these drugs, which leads to abnormal lipid peroxidation, accumulation of toxic metabolites, and death of hepatocytes. One of the main causes of programmed cell death of hepatocytes is ferroptosis caused by iron ion-dependent lipid peroxidation [15].

**Figure 1:**
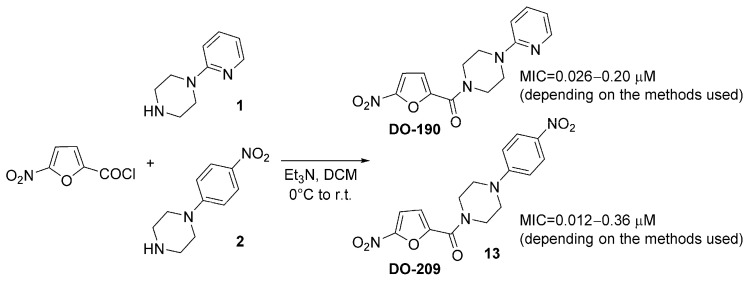
Synthesis of compounds **DO-190** and **DO-209** and their MIC values toward *Mycobacterium tuberculosis* H37Rv strain.

Here, we selected two nitrofurane derivatives (**DO-190** and **DO-209**), synthesized and described in our previous study (Figure 1), suitable for further investigation of their pharmacology and toxicology profile since they already demonstrated high in vitro antimycobacterial activity [14].

## 2. Materials and Methods

### 2.1. Chemistry

The synthesis, characterization, and in vitro antimycobacterial activity of **DO-190** and **DO-209** against *M. tuberculosis* H37Rv strain was described in detail in our previous study [14]. For purposes of this study, both compounds were prepared on a multigram scale.

### 2.2. Experimental Animals

The Animal Care Ethic Committee approved the study protocol, and Ethical clearance for the study was issued by the Bulgarian Agency for Food Safety (No 125 from 7 October 2020). The mice were housed, maintained, and euthanized following the relevant international rules and recommendations as stated in the European Convention for the Protection of Vertebrate Animals used for Experimental and Other Scientific Purposes (ETS 123) [18].

Male and female pathogen-free Jcl: ICR mice (6 weeks old, 25–30 g) obtained from the National Breeding Center, Sofia, Bulgaria, were used in the experiments. As a more sensitive sex, 48 females were used in the acute toxicity test and 36 males in the short-term toxicity test [19]. Mice were housed in Plexiglas cages (6 per cage) in a 12/12 light/dark cycle under standard laboratory conditions (ambient temperature 20 ± 2 °C and humidity 72 ± 4%). The standard complete commercial pelleted mice feed suitable for their age and fresh drinking water were available *ad libitum* during the entire experimental period. Before the start of the experiment, mice were acclimatized to vivarium conditions for seven days, and their health was monitored daily.

#### 2.2.1. Acute Toxicity in Mice

Acute toxicity was assessed in 48 female mice after peroral (p.o.) and intraperitoneal (i.p.) administration of the compounds using a simplified method of Lorke with slight modifications [20]. Three animals were used per dose at 5 fixed-dose intervals. For compound **DO-209,** the highest dose for both routes of administration was 3000 mg/kg, while 500 mg/kg was the lowest for i.p. administration. For compound **DO-190,** the highest dose used for the acute toxicity test was 1000 mg/kg for oral and i.p. application, and 200 mg/kg was the lowest dose used for both ways of administration. Due to the low water solubility of both investigated compounds, they were solubilized with Tween 80 (0.1%) before application.

The *LD*_50_ was calculated using the following equation: ${LD}_{50}=\sqrt{\left(D_{0}\times D_{100}\right)}$, where *D*_0_ is the highest non-lethal dose and *D*_100_ is the lowest lethal dose [21].

Surviving animals were observed every 3 h for the first 24 h and once a day for up to 14 days. During this period, the behavior of animals and the basic activities related to their breeding (walking, running, climbing, wrestling, and social behavior in a cage) were observed. Food intake and water intake were monitored daily Animals’ responses to “external stimuli” (manipulation response, straightening reflex, clapping response, response to noise or light fluctuations, toe, or tail squeezing reflex) were assessed. On day 14, animals were euthanized after anesthesia with ketamine/xylazine (80/10 mg/kg, i.p.), and an examination of the internal organs for possible macroscopic abnormalities (organ color, consistency, neoplasms, etc.) was done.

#### 2.2.2. Sub-Acute Toxicity

The sub-acute toxicity effects were assessed after repeated (14 days) oral administration to male mice. Based on the LD_50_ value after oral administration of **DO-209** (higher than 2500 mg/kg), two doses of 125 mg/kg and 250 mg/kg (≈1/20 and 1/10 of the LD_50_) were selected for multiple administrations. The second compound, **DO-190,** was applied at doses 35 and 70 mg/kg, based on the LD_50_ ≈ 700 mg/kg for oral administration. The experiments were performed with male Jcl: ICR mice at the same age of 6 weeks and weighing approximately 30–35 g, in which the substances were administered daily for 14 days orally with a gastric tube at approximately the same time of the day. Animals were observed daily for behavioral changes and signs of toxicity. All results were compared with the positive control ethambutol (EMB).

#### 2.2.3. Experimental Design

For the sub-acute toxicity tests, the animals were divided into 6 experimental groups of 6 mice in each. Group 1—control mice; Group 2—mice treated orally with EMB 50 mg/kg [22], Group 3—mice treated orally with **DO-190** at a dose of 35 mg/kg (1/20 LD_50_); Group 4—mice treated orally with **DO-190** at a dose of 70 mg/kg (1/10 LD_50_); Group 5—mice treated orally with **DO-209** at a dose of 125 mg/kg (1/20 LD_50_); Group 6—mice treated orally with **DO-209** at a dose of 250 mg/kg (1/10 LD_50_).

The weight of the experimental animals was measured with a laboratory balance on days 1, 3, 5, 7, 9, 11, and 13. On day 14, the animals were anesthetized with ketamine/xylazine and decapitated. Blood hematological parameters were assessed as follows. White blood cell number, red blood cell count, platelet count, hemoglobin concentration, and hematocrit were measured using commercial kits for a semi-automated hematological analyzer (BC-2800 Vet, Mindray, Shenzhen, China) following the instructions of the manufacturer. Serum biochemical parameters were assessed as follows. Blood glucose levels, urea, creatinine, uric acid, total protein, albumin, aspartate aminotransferase, alanine aminotransferase, total bilirubin, and direct bilirubin were measured using commercial kits for automated biochemical analyzer (BS-120, Mindray, China) following the instructions of the manufacturer. Blood, livers, and lungs were taken to assess oxidative stress and antioxidant status in the study groups. Livers, kidneys, and small intestines were taken for histological analysis.

### 2.3. Pathomorphological Evaluation of Tissue Specimens

Tissues from the liver, kidney, and small intestines of the mice from all groups were collected postmortem and fixed in 10% buffered formalin for 48 h. Fixed tissues were processed according to the classical paraffin method [23]. The cutting of the paraffin blocks was performed using a paraffin rotary microtome Leica RM 2255 at a slice thickness of 5 µm. The sections were stained with hematoxylin and eosin (H&E). Histological changes were examined and imaged with a Leica DM2500 light microscope equipped with a Leica MC120HD digital camera and Euromex BioBlue (Belgium) digital camera.

### 2.4. Biochemical Markers of Oxidative Stress Determination

#### 2.4.1. Lipid Peroxidation Inhibition Assay

As a marker of lipid peroxidation for the formation of endogenous lipid peroxidation products, malondialdehyde (MDA) was tested. The method is based on the reaction of MDA with 2-thiobarbituric acid (TBA) to the end products of lipids oxidation—thiobarbituric acid reactive substances (TBARS), which are detected spectrophotometrically (λ_max_ = 532 nm). The experimental procedure was adapted by Mileva et al. [24]. Briefly, the spontaneous lipid peroxidation in postnuclear homogenates of the liver, normalized to a final concentration of 2 mg protein/mL was diluted in 0.15 M KCl–10 mM potassium phosphate buffer, pH 7.2. The incubation media was heated for 15 min at 100 °C in the presence of 40% TCA + 5N HCl + 2% thiobarbituric acid (2:1:2 *v*/*v*) for color development. After cooling and centrifugation, the absorbance was read at 532 nm against dd H_2_O as a blank. The values are expressed as nmoles malondialdehyde nmol MDA per mg protein, using the molar extinction coefficient of 1.56 × 10^−5^ M^−1^cm^−1^.

#### 2.4.2. Measurement of the Total Glutathione

The method of Rahman et al. [25] for the total glutathione (tGSH) concentration in tissue culture was used. The principle of the reaction is based on the interaction between GSH and 5,5′-dithiobis-2-nitrobenzoic acid (DTNB). The rate of formation of the colored substance, 5′-thio-2-nitrobenzoic acid (TNB), is proportional to the concentration of tGSH in the sample. The maximal absorption of colored TNB is at 412 nm. The concentration of tGSH was calculated using oxidized glutathione as a reference standard and was expressed as ng/mg protein.

#### 2.4.3. Enzyme Activity of Superoxide Dismutase (SOD)

SOD activity was measured by the method of Beauchamp and Fridovich [26]. Superoxide anion radicals were generated photochemically for 7 min. As a result, the reduced nitro blue tetrazolium (NBT) was obtained as insoluble formazan in violet color. The decrease of NBT colorization in the presence of different enzyme concentrations was realized in a concentration-dependent manner. The changes of the absorptions were read at 560 nm vs. control of the reaction mixture. The amount of enzyme performing 50% inhibition of NBT reduction is accepted as a unit of activity (U/mg protein) [26].

#### 2.4.4. Measurement of the Glutathione Peroxidase Activity

The Glutathione Peroxidase Cellular Activity Assay Kit (Cat. No. CGP1) and Glutathione Reductase Assay Kit (Cat. No. GRSA) were used to measure the activities of glutathione-related enzymes.

#### 2.4.5. Protein Content

For the determination of the protein content, the Biuret method based on a colorimetric test for total protein was used. The Assay Kit was purchased from Cromatest/Lineal Chemicals REF №1153020.

### 2.5. Molecular Docking

Molecular docking studies were performed using Molecular Operating Environment developed by the Chemical Computing Group (MOE, https://www.chemcomp.com/MOE-Molecular_Operating_Environment.htm, version 2022.02, accessed on 21 June 2023). Docking simulations were carried out using the following X-ray crystallographic structures of *Mycobacterium tuberculosis*:

(1) The crystal structure of *M. tuberculosis* enoyl reductase (InhA) complexed with 5-hexyl-2-(2-methylphenoxy)phenol (TCU) with a co-factor nicotinamide adenine dinucleotide (NAD^+^), extracted from Protein Data Bank (http://www.rcsb.org/, PDB ID 2X22, accessed on 16 February 2023).
(2) The crystal structure of *M. tuberculosis* InhA complexed with (3s)-1-cyclohexyl-n-(3,5-dichlorophenyl)-5-oxopyrrolidine-3-carboxamide (ligand ID 641, further denoted as 641), also with a co-factor NAD^+^, extracted from PDB (PDB ID 4TZK).
(3) The crystal structure of *M. tuberculosis* galactofuranosyltransferase 2 (GlfT2) complexed with glycerol (Gol) and a co-factor uridine-5′-diphosphate (UDP), extracted from PDB (PDB ID 4FIY).
(4) The crystal structure of *M. tuberculosis* oxidoreductase complexed with ethanediol (Edo), extracted from PDB (PDB ID 4NXI).

As already mentioned, there is a limited number of nitrofuranyl compounds tested as anti-tubercular agents, which makes the task of choosing a target especially hard. The choice of the aforementioned targets has been partially inspired by the previous study. Ortiz et al. [27] examined galactofuranosyltransferase 2 (GlfT2) as an enzyme involved in the biosynthesis of *Mycobacterium tuberculosis* cell wall, thus attracting great interest in it, and presented thorough in silico analysis of its potential inhibitors. Ahsan et al. [28] presented novel 1,3,4-oxadiazole analogs as potent antimicrobial and anti-tubercular agents and directed their further investigation on possible action on enoyl reductase. Kumar et al. [29] considered nitrofuranyl amides a structural class of anti-tuberculosis agents, but with no docking studies performed. Geng et al. [30] presented results from docking studies but for nitrofuranyl calanolides.

Before proceeding to docking, water molecules were removed from the crystal structures, while the co-factors NAD^+^ and UDP were kept. For the positioning of the missing hydrogen atoms, the “Protonate 3D” tool of MOE was used to assign the correct ionization states to the protein structure. Docking studies have been carried out in the “Docking” module in MOE. Docking was performed applying two protocols—in a rigid receptor and in an induced fit mode. In both protocols, a triangle matcher was used for placement and London dG for rescoring 1 stage, while keeping the top 30 ranked poses and with no refinement performed afterward. For further analysis of the molecular docking results, the “Ligand Interactions” MOE tool was used to visualize the protein–ligand interactions in the active site of the complexes.

## 3. Results and Discussion

### 3.1. Chemistry

The series of six new nitrofuranylamides were synthesized, purified, and characterized in our previous study [14]. Three compounds demonstrated significant in vitro activity toward *M. tuberculosis* H37Rv. One active compound was used for in vitro selection of spontaneous resistant mutants to reveal indirectly its possible mechanism of action. The other two active compounds (**DO-190** and **DO-209**) are the subject of this study (Figure 1).

### 3.2. Acute Toxicity in Mice

Dose intervals and symptoms observed after oral and intraperitoneal administrations of both investigated compounds **DO-190** and **DO-209** are presented in Table 1, Table 2 and Table 3.

Based on the results, the LD_50_ for **DO-190** was calculated as follows: $LD50=\sqrt{D0\times D100}$ = $\sqrt{600\times 400}$ = 489.89; LD_50_ ≈ 500 mg/kg for i.p. application.

Based on the results, the LD_50_ for **DO-190** was calculated as follows: $LD50=\sqrt{D0\times D100}$ = $\sqrt{800\times 600}$ = 692.82; LD_50_ ≈ 700 mg/kg for p.o. administration.

The doses used for the 14-day subacute oral toxicity are:

1/10 LD_50_ = 70 mg/kg; 1/20 LD_50_ = 35 mg/kg

Based on the results, the LD_50_ for **DO-209** was calculated as follows:

$\mathrm{LD}50\;=\sqrt{D0\;\times \;D100}$ = $\sqrt{2000\times 3000}$ = 2449.49, i.e., LD_50_ > 2000 mg/kg for i.p. application.

No mortality was observed with oral administration at the highest dose of 3000 mg/kg, i.e., LD_50_ > 2500 mg/kg.

For the sub-acute 14-day oral treatment, the doses used were as follows:

1/10 LD_50_ = 250 mg/kg and 1/20 LD_50_ = 125 mg/kg

**DO-190** exhibitsed more pronounced toxicity compared to **DO-209**, and **DO-190** exhibited its toxic effects at a dose of 1000 mg/kg, when 100% of parenterally treated and 67% of orally treated animals died of respiratory disturbances and ataxia.

In contrast, **DO-209** administered at relatively higher doses did not result in death by either route of application, except the dose of 3000 mg/kg, administered intraperitoneally, where only one of three animals died. According to the Hodge and Sterner scale [31] **DO-209** could be classified as a slightly toxic compound.

Animals that survived the acute toxicity tests were monitored once daily for 14 days. During this period, no changes were observed in the social behavior in the cage and the animals’ responses to ‘external stimuli’. No changes were noted in skin, coat, eyes, mucous membranes, secretion, and autonomic activity (lacrimation, piloerection, changes in pupil size, or abnormal respiratory movements). No changes in gait or response to handling were observed, as was the presence of clonic or tonic movements or odd behavior (e.g., aggression, walking backward). No neurotoxic effects, such as sensory reactivity to different types of stimuli (auditory, visual, and proprioceptive), were reported. No change in the amount of food and water intake was observed. On the 14th day after acute toxicity, all surviving animals were euthanized.

Macroscopic examination at autopsy showed no gross visible changes or lesions in vital organs. There were no changes in the size, weight, color, or consistency of the lungs, liver, heart, kidneys, stomach, spleen, or intestines. No abnormalities were found in the morphology of the gonads and brain.

### 3.3. Sub-Acute Toxicity of **DO-190** and **DO-209** after 14 Days of Oral Administration of the Tested Compounds

**Figure 2:**
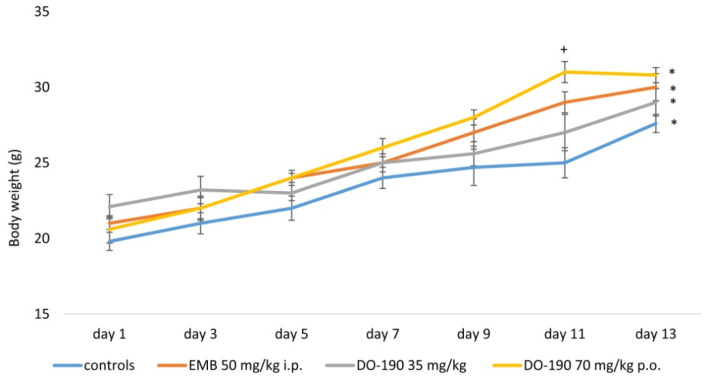
Changes in the body weight of animals treated with EMB, **DO-190**; ^+^ *p* < 0.05 vs. control; * *p* < 0.05 vs. first day of the experimental period.

**Figure 3:**
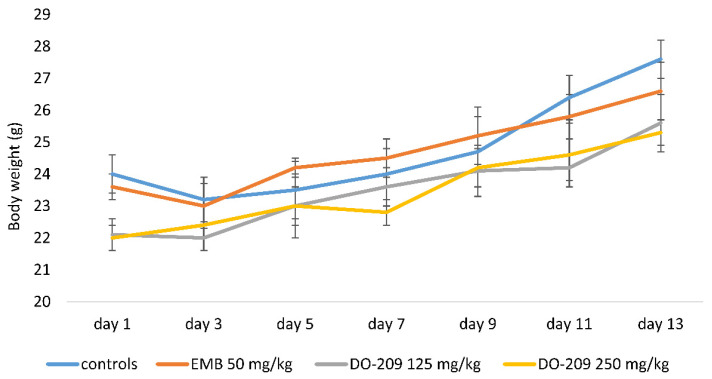
Changes in the body weight of animals treated with EMB, **DO-209**.

Changes in animal body weight during the 14-day experimental period are shown in Figure 2 and Figure 3.

During the 14-day experimental period, animals from all groups gained body weight, and this was observed to the greatest extent in mice treated with **DO-190** at a dose of 70 mg/kg. At the end of the experiment, this group reported a statistically significant increase in weight by 49.5% compared to the beginning of the experiment. In the groups treated with 35 mg/kg **DO-190** and ethambutol (EMB) 50 mg/kg, weight gain was 29% and 30%, respectively.

Over the whole period, stereotypies (repeating circles on the lid (grid) of the cage) were observed after the administration of **DO-190** at the higher dose of 70 mg/kg.

Administration of **DO-209** was accompanied by a lower weight gain of about 13–16% compared to the first day.

#### Complete Blood Count (CBC) and Biochemistry in the Blood of Mice

Table 4 and Table 5 summarize the results related to the blood count analysis of the experimental animals. Repeated administration of the test substances was associated with a slight increase in the number of WBC, especially at the higher doses, compared to the control animals, but these changes remained within the reference values for the species. No deviations were observed in other hematological parameters compared to the control.

Slight changes were found in some serum biochemical parameters. After 14 days of treatment of mice with EMB at 50 mg/kg, there was a statistically significant increase in uric acid level by 63% compared to the control. Both doses of **DO-190** increased the levels of urea, uric acid, aspartate aminotransferase, alanine aminotransferase, and total bilirubin compared to the control animals and EMB-treated mice, but this increase was within the reference values, indicating that the test compound administered orally at these two doses did not result in statistically significant toxic effects.

Compound **DO-209** administered at the higher dose of 250 mg/kg increased blood glucose level by 42%, urea level by 92%, and ALAT activity by 52%, respectively, over the control group, but these changes were close to the reference values.

### 3.4. Pathomorphological Evaluation of Tissue Specimens

#### 3.4.1. Liver

**Figure 4:**
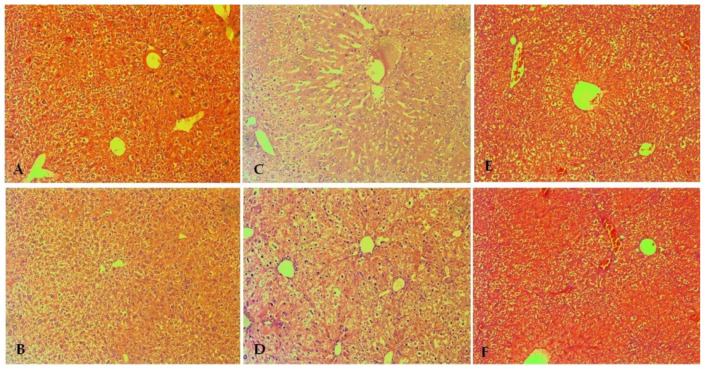
Pathomorphological findings in the liver of mice after oral administration of ethambutol (EMB) and nitrofuranes. Legend: (**A**) Control group—not treated; (**B**) EMB 50 mg/kg; (**C**) **DO-190** 35 mg/kg; (**D**) **DO-190** 70 mg/kg; (**E**) **DO-190** 800 mg/kg; (**F**) **DO-209** 3000 mg/kg. Magnification of the field 100×.

The pathological-morphological profile of the liver shows the absence of pathological changes. The microscopic findings in the liver of treated mice are presented in Figure 4. The organ parenchyma shows the lobular structure, absence of areas of remodeling, intrahepatic cholestasis in individual cells, and regenerative activity within tissue-specific parameters. Biliary parameters included properly presented structures and the absence of proliferative changes in the portal canal. Organ blood flow included passive venous hyperemia, dilatation of sinusoidal spaces, and preserved hepatic laminae. The great and middle organ veins had preserved histologic structure with no signs of intimal changes. Isolated intrahepatic cholestasis was found. The lobular inflammatory process and loss of hepatocytes were not reported in the treated groups with EMB at a dose of 50 mg/kg, and both compounds **DO-190** and **DO-209** at a dose of 35 mg/kg, 70 mg/kg, 800 mg/kg, 3000 mg/kg, respectively).

Signs of increased ballooning degeneration of hepatocytes were found in the range of 8–18%. Minimal small vesicular steatosis (less than 3%) was reported. The histological profile shows isolated degenerative changes in hepatocytes with a non-zonal distribution. The changes are not associated with toxic organ manifestations. Cytoplasmic changes and centrally located nuclei in intracytoplasmic septa were observed in some cells. The finding was not recurrent and was not accompanied by loss of membrane organelles. The possibility of metabolic overload of some types of membrane organelles was not accompanied by increased turnover of these organelles, and autophagic vacuoles were observed as a single finding.

In the oral and intraperitoneal **DO-209**-treated groups at a dose of 3000 mg/kg, lesions with increased balloon degeneration were observed in 2–12% and 2–14%, respectively. Signs associated with an initiated fibroblastic process (initiated organ collagenization) were found as an isolated finding of minimal perisinusoidal and portal collagenization in the oral and intraperitoneal **DO-209**—treated groups at a dose of 3000 mg/kg.

#### 3.4.2. Kidneys

**Figure 5:**
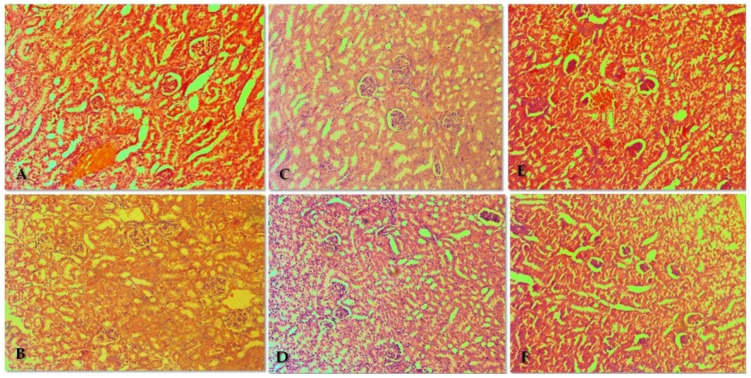
Pathomorphological findings in the kidney in mice after oral administration of EMB and nitrofuranes. Legend: (**A**) Control group—not treated; (**B**) EMB 50 mg/kg; (**C**) **DO-190** 35 mg/kg; (**D**) **DO-190** 70 mg/kg; (**E**) **DO-190** 800 mg/kg; (**F**) **DO-209** 3000 mg/kg. Magnification of the field 100×.

Changes in the kidneys did not show the topography of the organ poles. The microscopic findings in the kidneys of treated mice are presented in Figure 5. The histological findings include features consistent with normal histological architecture without being associated with concomitant pathological conditions. The vessel presentation was histologically consistent and was not accompanied by vascular fibrointimal changes and luminal reduction. There was no evidence of tubulitis, tubular atrophy, and glomerulitis.

The cortical labyrinth is correctly represented by different luminal zones in the proximal tubules at the height of the upright cells in the reference normal. The curved portions of the distal tubules have lower upholstered epithelium, wide luminal segments, and preserved histologic structure. Elements of the glomerular and extragllomerular mesangium are visualized in distinct areas and show no signs of proliferation. The straight segments of the proximal tubules are found in separate areas. The outer medullary zone shows a narrow variation in thickness in the organs of the exposed groups. Visualization of the inner zone shows collecting tubules with preserved histological structures. Minimal variation in the periglomerular interstitium presented. The excretory structures of the organs (renal sinuses and ampullary part of the renal pelvis) have preserved histological structure at the exposure doses. In control group A, the cortical labyrinth had different luminal areas in the proximal tubules at the height of the papillary cells in the reference normal.

On histological preparation B (EMB at a dose of 50 mg/kg), the curved portions of the distal tubules had lower papillary epithelium, broad luminal segments, and preserved histological structure. On histological preparation C (**DO-190** at a dose of 35 mg/kg), minimal circulatory lesions were observed, presented with edema in the interstitium. Histological preparation D (**DO-190** at a dose of 70 mg/kg) showed no tubulitis, tubular atrophy, or glomerulitis. On histological preparation E (**DO-190** at a dose of 800 mg/kg), the cortical labyrinth was correctly represented with different luminal areas. On histological preparation F (**DO-209** at a dose of 3000 mg/kg), minimal variations in the presented periglomerular interstitium were observed.

#### 3.4.3. Small Intestine

**Figure 6:**
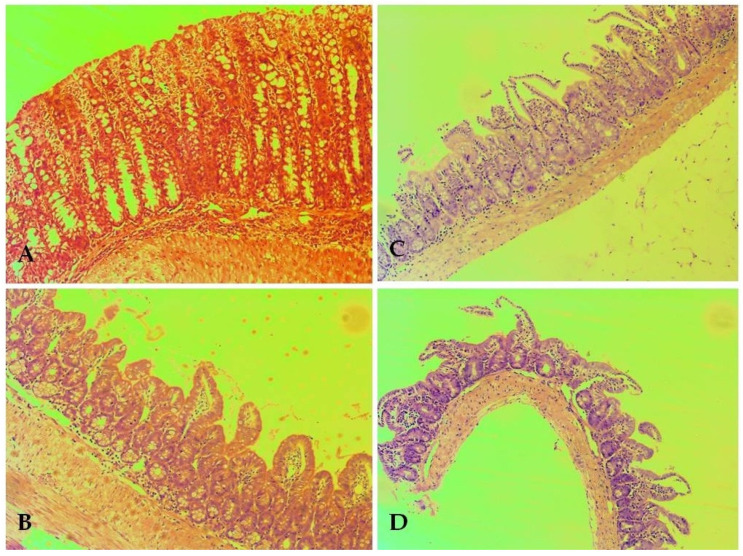
Pathomorphological findings in the small intestine of mice after oral administration of EMB and nitrofuranes. Legend: (**A**) Control group—untreated; (**B**) EMB 50 mg/kg; (**C**) **DO-190** 35 mg/kg; (**D**) **DO-190** 70 mg/kg. Magnification of field 100×.

Pathomorphological findings in the small intestine of mice after oral administration of EMB and nitrofuranyl amides are shown in Figure 6. In control group A, leukocytes did not show a high density. Histological preparation B (EMBat a dose of 50 mg/kg) showed no epithelial defects reaching and exceeding the muscular layer of the epithelium (lamina muscularis mucosae). Changes in crypt architecture were isolated. The ratio of intestinal villi to crypts was predominantly 2:1. This determined a score of 1 and a low (minimal) degree of villous obturation. Histological preparation C (**DO-190** at a dose of 35 mg/kg) showed a low degree of the inflammatory process. In histological preparation D (**DO-190** at a dose of 70 mg/kg), non-parallel crypts were found in each treatment group. Different diameters of crypts were found, such as a very low frequency of finding. Neutrophil leukocytes in the crypt lumen were single.

### 3.5. Markers of Oxidative Stress

For a complete pharmacological profile of both compounds and to elucidate the mechanisms of their toxicity, we studied the influencing ROS-mediated homeostasis in the liver of experimental animals.

As is well known, oxygen plays an essential role in the living processes of aerobic organisms, but it may become dangerous for them due to its ability to act as free radicals under stress conditions. A natural side effect of aerobic respiration is the generation of ROS [32]. Free radicals, e.g., reactive oxygen (ROS) and nitrogen (NOS) species are unstable, highly reactive structures, and their over-generation capable of triggering chain reactions resulting in damage to both cellular and extracellular macromolecules, such as proteins, lipids, and nucleic acids. The balance between the formation and elimination of free radicals determines the redox state and stability of the living organism, so understanding and controlling these interactions is essential to biological interactions [33]. The standard therapeutic scheme for the treatment of tuberculosis, which is recommended by the WHO, includes first-line drugs (isoniazid (INH) and ethambutol (EMB)) [34]. Therefore, when testing **DO-190** and **DO-209**, we used INH and EMB as control reference antibiotics.

The preclinical phase of drug development involves toxicity studies in animals. It should reveal potential toxicity that would occur at much lower therapeutic doses. These experiments usually detect potential hepatotoxicity inherent to the compound and allow the elimination of those related to the assessment of the risk of their administration, especially if they suffer from concomitant serious diseases. Hepatotoxicity induced by anti-tuberculosis drugs might result in significant morbidity and, rarely, even mortality [35]. In TB, oxidative stress is a result of tissue inflammation due to illness, as well as free radical bursts from activated macrophages. On the other hand, anti-tuberculosis drugs can induce free radical reactions and cause liver toxicosis during the treatment.

Although first-line anti-TB drugs are effective, their hepatotoxicity may lead to a higher rate of drug-induced liver toxicity. Fluoroquinolones have been used as second-line agents in the treatment of multidrug-resistant tuberculosis and cases of hepatotoxicity due to first-line agents. Quinolones are either metabolized in the liver (ciprofloxacin) or excreted unchanged by the kidneys (levofloxacin). Isoniazid, rifampicin, and pyrazinamide are reported to cause hepatotoxicity, while ethambutol and streptomycin are not hepatotoxic. Therefore, in our study, we used INH and EMB at their therapeutic doses as controls to compare the effects of the test compounds [36].

**Figure 7:**
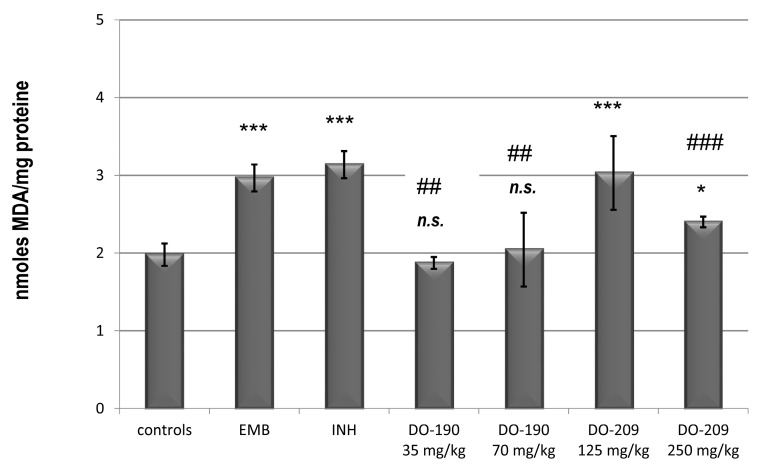
Endogenous content of MDA in the liver homogenate of experimental groups. * *p* < 0.05 vs. controls; *** *p* < 0.001 vs. controls; n.s*.—*nonsignificant vs. controls; ## *p* < 0.01 vs. EMB and INH; ### *p* < 0.001 EMB and INH. Results are expressed as mean ± SD (n = 6). The significance of the data was assessed using the nonparametric Mann–Whitney U test. Values of *p* ≤ 0.05 were considered statistically significant.

This form of toxicity potentially affects the outcome of tuberculosis treatment in more patients. The liver plays a critical role in the metabolism and detoxication of ingested and blood-borne substances. Many drugs, environmental toxicants, and selected dietary components have the potential to cause liver damage by inducing oxidative stress. The effect of newly synthesized compounds on oxidative damage in the liver was investigated. Figure 7 shows the changes in malondialdehyde (MDA) content as a biochemical marker of endogenous lipid peroxidation. Peroxidation of lipids in cells biomembranes is mediated by free radical reactions. It leads to membrane damage and has been proposed to be associated with the pathogenesis of tissue injuries [37]. MDA is an endogenous genotoxic product, considered a biochemical marker of enzymatic and/or ROS-mediated lipid peroxidation. MDA is the most popular indicator of oxidative damage to cells and tissues. It can lead to cross-linking polymerization of biological macromolecules and thus realize its damaging role as a serious cytotoxic and genotoxic factor. MDA content is usually used as a basis for evaluating the degree of lipid peroxidation and reflecting the level of damage to cells and tissues from the effects of pro-oxidant agents [38].

In the groups receiving both INH and EMB, the level of MDA increased by more than 35%. Doses of 35 and 70 mg/kg of compound **DO-190** did not cause significant deviations compared to the control animals (*p* < 0.001). The endogenous MDA content of compound **DO-209** at a dose of 125 mg/kg showed a comparable value with the INH and EMB controls, but at a dose of 250 mg/kg, the value was decreased.

According to Bains, glutathione (GSH) is considered to be the “master antioxidant” because it is the most-important redox regulator that controls inflammatory processes in the body [39]. Disturbances in GSH homeostasis have been associated with liver diseases induced by drugs, alcohol, diet, and environmental pollutants [40]. GSH can scavenge hydroxyl radicals and superoxide directly and serves as a co-factor for the enzyme glutathione peroxidase in metabolizing hydrogen peroxide as well as lipid peroxides. Furthermore, GSH can regenerate other important antioxidants, such as vitamins C and E, back to their active forms.

Therefore, glutathione level in the living organism is the best indicator of ongoing oxidative processes. GSH deficiency leads to the risk of oxidative damage to cells, and therefore, as expected, GSH imbalance was observed in a wide range of pathological conditions, including tuberculosis, HIV, diabetes, cancer, etc. When ROS production is not controlled, glutathione depletion occurs, rendering the patient susceptible to immunosuppression, organ damage, increased vascular permeability, shock, and thrombotic events [41].

**Figure 8:**
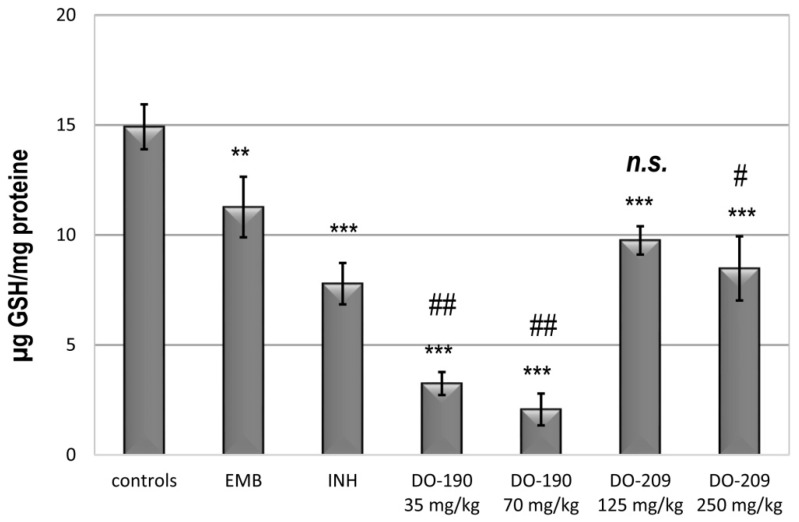
Endogenous content of GSH in the liver homogenate of experimental groups. *** *p* < 0.01 vs. controls; n.s.—nonsignificant vs. ** *p* < 0.01 vs control, # *p* < 0.01 vs EMB, ## *p* < 0.01 vs EMB and INH. EMB and INH. Results are expressed as mean ± SD (n = 6). The significance of the data was assessed using the nonparametric Mann–Whitney U test. Values of *p* ≤ 0.05 were considered statistically significant.

Significant dose-dependent decreases in the level of intracellular total glutathione were found in the **DO-190**-treated groups at both doses (Figure 8). Values were nearly 5-fold lower than those of untreated controls (*p* < 0.001), EMB (almost 3-fold at *p* < 0.01), and INH (>2-fold at *p* < 0.01). Administration of **DO-209** resulted in less reduction of glutathione compared to both drug-control animals and the EMB- and INH-treated groups. The correlation coefficient between MDA and glutathione levels was −0.838 for **DO-190** at a dose of 35 mg/kg, and 0.763 for **DO-190** at a dose of 70 mg/kg. The correlation coefficient between both levels was—0.093 for **DO-209** at a dose of 125 mg/kg, and 0.997 for **DO-209** at a dose of 250 mg/kg, respectively. Glutathione supplementation also significantly reduced the level of lipid peroxidation and the risk of liver damage [15].

**Figure 9:**
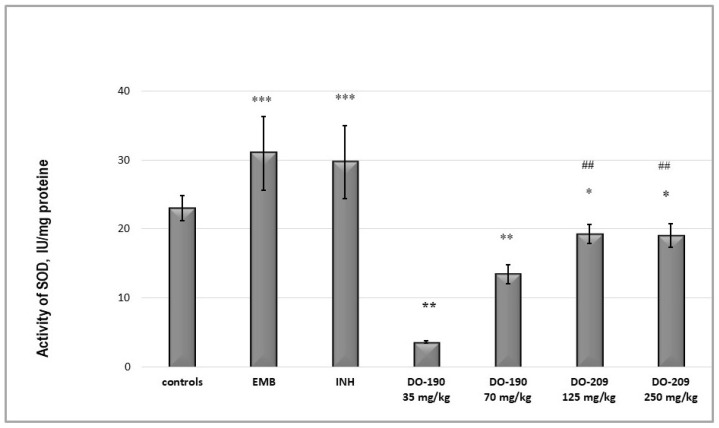
The activity of SOD in the liver supernatant of experimental groups. *** *p* < 0.001 vs. controls; ** *p* < 0.01 vs controls; * *p* < 0.05 vs. controls; ## *p* < 0.01 vs EMB and INH. Results are expressed as mean ± SD (n = 6). The significance of the data was assessed using the nonparametric Mann-Whitney U test. Values of *p* ≤ 0.05 were considered statistically significant.

Superoxide dismutase (SOD) is a metalloenzyme that is on the front line of defense against ROS-mediated injury [42]. It catalyzes the dismutation of superoxide anion free radical (O_2_^−^) into molecular oxygen and hydrogen peroxide (H_2_O_2_) and decreases the O_2_^−^ level, which damages the cells in conditions of oxidative stress [43]. In the animals receiving INH and EMB, an increased activity of SOD was observed, while at **DO-190** in both doses, the activity decreased significantly (*p* < 0.001), and at **DO-209,** it was quite weak compared to the controls (Figure 9).

Glutathione peroxidase (GPx) is the general name of an enzyme family with peroxidase activity whose central biological role is to protect the cells from oxidative damage [44]. On the biochemical level, glutathione peroxidase function is to reduce lipid hydroperoxides to their corresponding alcohols, as well as to reduce free hydrogen peroxide to water [45].

**Figure 10:**
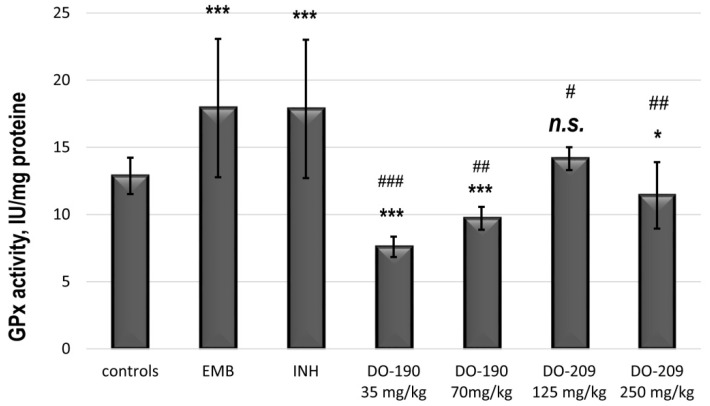
The activity of glutathione peroxidase in the liver supernatant of experimental groups. *** *p* < 0.001 vs. controls; * *p* < 0.05 vs controls; # *p* < 0.05 vs. INH 50 mg/kg; ## *p* < 0.01 vs. INH 50 mg/kg; ### *p* < 0.001 vs INH 50 mg/kg; n.s.—nonsignificant vs. controls. Results are expressed as mean ± SD (n = 6). The significance of the data was assessed using the nonparametric Mann–Whitney U test. Values of *p* ≤ 0.05 were considered statistically significant.

Figure 10 shows the results of GPx activities in the experimental groups. The INH and EMB increased GPx activity compared to the controls, while **DO-190** at both doses decreased it (*p* < 0.001). Reduced activity was also observed with **DO-209** administered at a higher dose of 250 mg/kg. In the mice receiving 250 mg/kg, the activity showed a slight reduction compared to the controls (*p* < 0.05) and was very noticeable compared to the EMB and INH-treated groups (*p* < 0.01).

The results presented in this study show that compound **DO-190** in both administered doses causes depletion of intracellular glutathione (Figure 8), accompanied by compensatory changes in the activities of SOD and GPx enzymes (Figure 9 and Figure 10). Probably, these changes were able to preserve the cell membranes from a process of lipid peroxidation and the formation of high levels of MDA as a biochemical marker for this process (Figure 7). Rafique et al. [46] reported a study that described the significant hepatoprotective potential of plant extracts rich in polyphenols and flavonoids against INH-induced hepatotoxicity in male mice model. Flavonoids and polyphenol compounds have a strong antioxidant activity, which has a hepatoprotective role against free radical injury. In our study, the two compounds **DO-190** and **DO-209** disturb the oxidative balance in mouse liver. Further investigations would contribute to a more precise elucidation of the mechanisms of possible hepatoprotection.

### 3.6. Molecular Docking

Molecular docking studies of two investigated compounds, as well as of EMB, were performed in the four specified targets, firstly applying docking in rigid receptors. The obtained scores after docking placed EMB in the favorable position toward the investigated compounds in three out of four chosen targets (Compounds **DO-190** and **DO-209** outperformed EMB only in the active site of InhA (PDB IDs 2 X22). This fact provoked the idea of docking studies to be performed in the induced fit mode. In addition, INH was subjected to docking as an important anti-TB drug. Table 6 presents the scores after docking performed in the four specified targets in induced fit mode for the investigated compounds along with EMB and INH.

Both compounds **DO-190** and **DO-209** demonstrated promising results from the point of view of docking scores, showing binding energies lower than those by EMB in the active sites of InhAs (PDB IDs 2 X22 and 4TZK). In the case of GlfT2 (PDB ID 4FIY), the docking score of EMB placed it between two investigated compounds, while in the case of oxidoreductase (PDB ID 4NXI), EMB outperformed both **DO-190** and **DO-209**. Concerning the docking scores of INH, the investigated compounds showed preferable energies in three out of four targets, except for the oxidoreductase (PDB ID 4NXI). The docking scores of the second-best pose of **DO-209** after docking in the active site of InhA (PDB ID 4TZK) and the fourth-best pose of EMB after docking in the active site of InhA (PDB ID 2 X 22) are listed instead of the best scores since they are the first ones that demonstrated protein–ligand interactions. **DO-190** showed preferable binding energies compared to the **DO-209** in three out of four targets, except the InhA (PDB ID 2 X 22).

In addition to the analysis based on binding energies (Table 6), an analysis of the protein–ligand interactions (PLIs) was also performed. EMB was used as a reference control drug.

**Figure 11:**
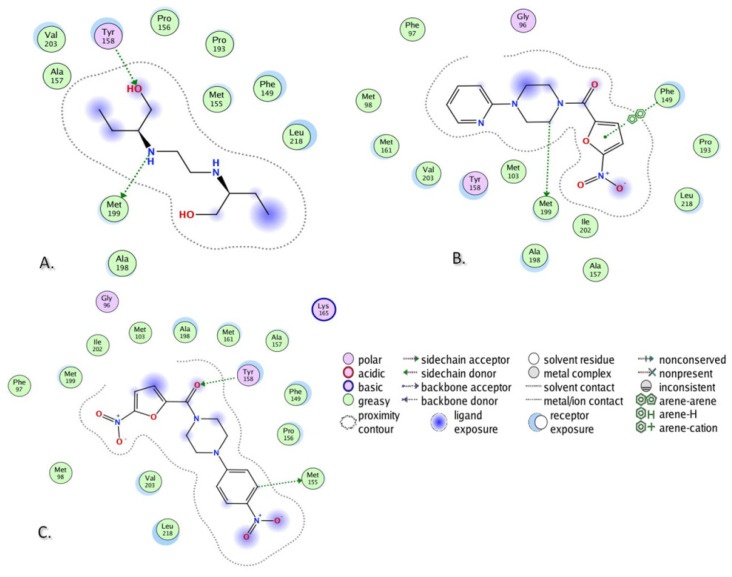
PLI of (**A**) EMB, (**B**) **DO-190,** and (**C**) **DO-209** in the ligand-binding domain of InhA (PDB ID 2X22).

The results are presented in Table 7. In the case of InhA (PDB ID 2 X 22), EMB demonstrated two PLIs: Two H-bonds with Tyr158 and Met199 (Figure 11A). Compound **DO-190** repeated the H-bond with Met199, shown by the EMB, while it demonstrated a newly recognized PLI, arene–arene interaction with Phe149 (Figure 11B). Compound **DO-209** repeated the H-bond with Tyr158, shown by EMB, and demonstrated a newly recognized PLI—H-bond with Met155 (Figure 11C). For both compounds, Tyr158 in the case of **DO-190**, and Met199 in the case of **DO-209**, are at the receptor exposure, very close to the ligand, but still not in a binding distance.

**Figure 12:**
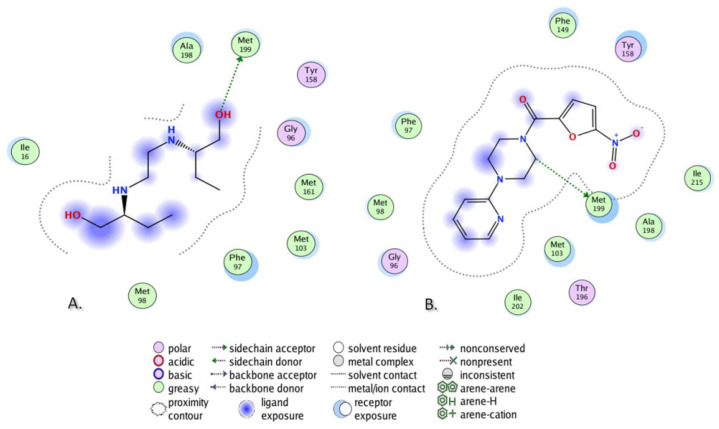
PLIs of (**A**) EMB and (**B**) **DO-190** in the ligand-binding domain of InhA (PDB ID 4TZK).

In the case of another structure of InhA (PDB ID 4TZK), EMB demonstrated an H-bond with Met199 (Figure 12A). Compound **DO-190** repeated the PLI, shown by the EMB (Figure 12B), while **DO-209** demonstrated a newly recognized H-bond with Tyr158 For **DO-209**, Met199 is at the receptor exposure, very close to the ligand, but still not in a binding distance.

**Figure 13:**
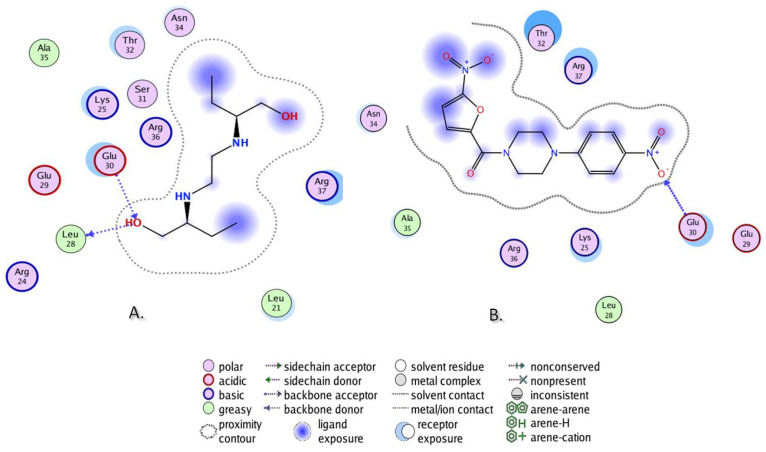
PLIs of (**A**) EMB and (**B**) **DO-209** in the ligand-binding domain of GlfT2 (PDB ID 4FIY).

In the case of GlfT2 (PDB ID 4FIY), EMB demonstrated two H-bonds—with Leu28 and Glu30 (Figure 13A). Compound **DO-209** repeated the H-bond with Glu30 (Figure 12B), shown by the EMB, while **DO-190** demonstrated a newly recognized H-bond with Arg37). For **DO-190**, Leu28 and Glu30 are at the receptor exposure, very close to the ligand, but still not in a binding distance.

In the case of oxidoreductase (PDB ID 4NXI), none of the investigated compounds repeated the H-bond with Asp12, which was demonstrated by EMB. Meanwhile, both compounds **DO-190** and **DO-209** revealed newly recognized H-bonds with Asn41 and Gly160, while **DO-209** showed one more H-bond with Gly7). For both compounds, Asp12 is at the receptor exposure, very close to the ligand, but still not in a binding distance. PLIs analysis demonstrated that both compounds **DO-190** and **DO-209** repeated some of the specific PLIs shown by EMB in three out of four chosen targets.

## 4. Conclusions

In the present study, we assess the toxicity and oxidative stress of two recently synthesized nitrofuranyl amides—**DO-190** and **DO-209,** with high in vitro antimycobacterial activity. In addition, molecular docking models were applied to both compounds to elucidate the possibilities for further development of new anti-tuberculosis candidates with improved efficacy, selectivity, and pharmacological parameters. Acute toxicity tests showed that no changes were observed in the skin, coat, eyes, mucous membranes, secretions, and vegetative activity in mice (lacrimation, piloerection, changes in pupil size, or abnormal respiratory movements). Neurotoxic effects, such as sensory reactivity to various types of stimuli (auditory, visual, and proprioceptive), were not observed. There was no change in the amount of food and water intake. The histological findings include features consistent with normal histological architecture without being associated with concomitant pathological conditions. Weight gain and lack of statistically significant changes in hematological, biochemical, and pathomorphological parameters in the blood, liver, small intestines, and kidney of mice treated for 14 days with two compounds indicate good tolerance of the experimental animals to them. The observed oxidative stress markers indicated that both compounds disturbed the oxidative balance in the mouse liver. Further research would contribute to a more complete elucidation of the mechanisms by which a possible hepatoprotection would occur. Based on the molecular docking, **DO-190** and **DO-209** in the four specified targets in comparison to those of the EMB, demonstrated potential protein–ligand interactions and could be considered for further investigations. Thus, the studied compounds exhibit promising activity with low toxicity and could be considered lead compounds for the development of antimycobacterial agents.

## Figures and Tables

**Table 1:** Acute intraperitoneal toxicity of **DO-190.**

Dose mg/kg	Lethality	Time of Appearance	Symptoms before Lethal Outcome
1000	3/3 (100%)	15–30 min	Rapid breathing, ataxia, piloerection
800	2/3 (67%)	After 0.5 h	Difficulty of breathing, ataxia
600	1/3 (33%)	After 1 h	Difficulty of breathing, ataxia
400	0/3	-	-
200	0/3	-	-

**Table 2:** Acute oral toxicity of **DO-190**.

Dose mg/kg	Lethality	Time of Appearance	Symptoms before Lethal Outcome
1000	2/3 (67%)	15–30 min	Rapid breathing, ataxia, piloerection
800	1/3 (33%)	After 2 h	Difficulty breathing, ataxia
600	0/3	-	-
400	0/3	-	-
200	0/3	-	-

**Table 3:** Acute intraperitoneal toxicity of **DO-209.**

Dose mg/kg	Lethality	Time of Appearance	Symptoms before Lethal Outcome
3000	1/3 (33%)	-	Rapid breathing, ataxia, piloerection
2000	0/3	-	-
1500	0/3	-	-
1000	0/3	-	-
500	0/3	-	-

**Table 4:** Complete blood count (CBC) after 14 days of administration of EMB and nitrofuranes: EMB 50 mg/kg (EMB), **DO-190** at doses 35 mg/kg (A) and 70 mg/kg (B); **DO-209** at doses 125 mg/kg (C) and 250 mg/kg (D).

CBC	Control	EMB	A	B	C	D	Ref. Values
WBC x 10^9^/L	6.4 ± 0.5	7.8 ± 0.6 ^a^	7.1 ± 0.7	8.23 ± 0.13 ^a^	6.8 ± 0.6	8.4 ± 0.28 ^a^	2.9–15.3
RBC x 10^12^/L	7.06 ± 0.4	6.36 ± 0.8	7.03 ± 0.5	6.02 ± 0.2	7.23 ± 0.7	7.12 ± 0.4	5.6–7.89
Hgb g/L	142 ± 7.2	142 ± 2.6	136 ± 3.2	128 ± 4.1	135 ± 2.4	139 ± 4.2	120–150
HCT %	44 ± 2.4	41 ± 3.2	42.4 ± 2.2	43.2 ± 3.1	42.2 ± 3.7	43.2 ± 2.1	36–46
PLT 10^9^/L	789 ± 96	881 ± 123	865 ± 105	932 ± 116	888 ± 121	787 ± 212	100–1610

^a^ *p* < 0.05 vs. controls; vs. EMB. Results are expressed as mean ± SD (*n* = 6). The significance of the data was assessed using the nonparametric Mann–Whitney U test. Values of *p* ≤ 0.05 were considered statistically significant. Abbreviations: WBC (white blood cells); RBC (red blood cells); Hgb (hemoglobin); HCT (hematocrit), PLT (platelets).

**Table 5:** Biochemical parameters (BP) of serum from experimental animals after 14 days of administration of EMB and nitrofuranes: EMB 50 mg/kg, **DO-190** at doses 35 mg/kg (A) and 70 mg/kg (B); **DO-209** at doses 125 mg/kg (C) and 250 mg/kg (D).

BP	Control	EMB	A	B	C	D	Ref. Values
GLU mmol/L	6.2 ± 0.12	7.1 ± 0.4	6.3 ± 0.5	7.3 ± 0.32	6.5 ± 0.41	8.8 ± 0.29 ^ab^	4.2–7.5
UREA mmol/L	7.1 ± 0.32	8.0 ± 0.36	11.4 ± 0.31 ^ab^	12.6 ± 0.28 ^ab^	6.8 ± 0.22	13.6 ± 0.22 ^ab^	3.27–12.1
CREAT µmol/L	88 ± 12.3	82 ± 12.8	92.3 ± 8.2	102.2 ± 11.6	85 ± 16.2	79 ± 12.6	35–120
UA µmol/L	236 ± 11.4	385 ± 20.3 ^a^	286.3 ± 9.1 ^ab^	298 ± 11.3 ^ab^	243 ± 13.3	261 ± 12.3	0–300
TP g/L	58 ± 3.2	53 ± 4.6	55.2 ± 2.8	58.1 ± 3.1	54 ± 5.3	56 ± 3.3	53–63
ALB g/L	27 ± 1.3	28 ± 1.2	27.2 ± 0.8	26.8 ± 1.6	26 ± 2.2	27 ± 3.1	26–29
ASAT U/L	83 ± 4.1	86 ± 5.4	121.2 ± 3.2 ^ab^	122.2 ± 2.1 ^ab^	91 ± 3.8	98 ± 4.8	65–122
ALAT U/L	58 ± 4.2	59 ± 6.1	80.2 ± 2.2 ^ab^	79.3 ± 1.3 ^ab^	62.2 ± 4.3	88.4 ± 3.6 ^abc^	55–80
T-Bil µmol/L	5.6 ± 0.48	6.0 ± 0.28	8.2 ± 0.41 ^ab^	8.4 ± 0.8 ^ab^	4.4 ± 0.42	6.3 ± 0.38	3.9–9.6
D-Bil µmol/L	3.4 ± 0.86	3.5 ± 0.84	3.8 ± 0.31	4.1 ± 0.61	3.4 ± 0.56	4.9 ± 0.24	0–6.8

^a^ *p* < 0.05 vs. controls; ^b^ *p* < 0.05 vs. EMB; ^c^ *p* < 0.05 vs. Reference values; Results are expressed as mean ± SD (*n* = 6). The significance of the data was assessed using the nonparametric Mann–Whitney U test. Values of *p* ≤ 0.05 were considered statistically significant. Abbreviations: GLU (glucose level); CREAT (creatinine); UA (uric acid); TP (total protein); ALB (albumin); ASAT (aspartate aminotransferase); ALAT (alanine aminotransferase); T-Bil (total bilirubin); D-Bil (direct bilirubin).

**Table 6:** Docking scores of the investigated compounds in the active sites of InhA (PDB ID 2X22 and 4TZK), GlfT2 (PDB ID 4FIY), and oxidoreductase (PDB ID 4NXI).

Compounds	Docking Score *			
	2 X 22	4TZK	4FIY	4NXI
**DO-190**	−11.65	−11.73	−9.20	−7.81
**DO-209**	−11.77	−11.67	−8.50	−6.73
EMB	−9.63	−10.73	−8.75	−8.45
INH	−9.69	−9.70	−7.79	−8.90

* E_score 1—free binding energy from the first rescoring stage, in kcal/mol.

**Table 7:** Visual inspection of the protein–ligand interactions (PLI) diagrams.

Compounds	Protein–Ligand Interactions			
	2X22	4TZK	4FIY	4NXI
**DO-190**	Phe149 Met199	Met199	Arg37	Asn41 Gly160
**DO-209**	Met155 Tyr158	Tyr158	Glu30	Gln7 *Asn41* *Gly160*
EMB	Tyr158 Met199	Met199	Leu28 Glu30	Asp12

## Data Availability

All obtained data are presented in this article.
